# Surgical treatment of depression fractures of the lateral tibial plateau using porous titanium granules

**DOI:** 10.1080/03009730802579752

**Published:** 2009-02-04

**Authors:** Brynjólfur Jónsson, Bengt Mjöberg

**Affiliations:** ^1^Malmö University HospitalMalmöSweden; ^2^Tigran Technologies AB, Medeon Science ParkMalmöSweden

**Keywords:** Titanium, biocompatible Materials/*therepeutic use, bone graft substitutes, tibial fractures, pilot study, clinical study

## Abstract

The clinical and radiological results were excellent in this pilot study of four cases of depression fracture of the lateral tibial plateau, where, in addition to internal fixation with screws or a buttress plate, porous titanium granules were used to support the elevated articular surface.

## Introduction

In the surgical treatment of depression fractures (Schatzker II–III) of the lateral tibial plateau (which constitute more than 50% of all tibial plateau fractures) the goal is reduction of the articular surface and stable fixation. The subchondral defect in the metaphysis is usually grafted with bone from the iliac crest to support the elevated articular surface. Disadvantages are pain symptoms (1) and other donor site complications (2), as well as resorption of the graft with subsequent loss of reduction. Non-resorbable, osteoconductive bone substitutes may therefore be an advantage over autogenous bone grafts. In this pilot study we used titanium granules—which previously have been used to stabilize hip prostheses (3,4) and to enhance bone regeneration (5)—as bone substitute.

## Patients

Four patients, 49–62 years old (two in Ystad 2004 and two in Malmö 2007), were treated surgically for depression fracture (Schatzker II–III) of the lateral tibial plateau using porous titanium granules of 1–1.4 mm in size (Ortix™, Tigran Technologies AB, Malmö, Sweden) in addition to internal fixation with screws or a buttress plate. The two Ystad-patients were allowed to exercise directly after surgery, while the two Malmö-patients were put in plaster for 6 weeks. Postoperatively, partial weight-bearing was allowed, but full weight-bearing was not allowed until 6 weeks after surgery.

The surgical facilities were improved during the pilot time period, including a suitable drill bit to fenestrate the cortex beneath the lateral tibial plateau in pure depression fractures through which the depressed articular surface can be elevated. A syringe fit for injecting titanium granules through the fenestration and a titanium net for sealing the fenestration were also produced.

## Results

A 61-year-old man with a pure depression fracture was pain-free 6 weeks after surgery and walked perfectly without any kind of support. As he did not live in the neighbourhood of Ystad, he was reluctant to participate in further controls. A 49-year-old woman with a pure depression fracture had slight discomfort 6 weeks after surgery, returned to her previous employment after 2.5 months, and was pain-free after 6 months. A 62-year-old man with a split depression fracture returned partially to his previous employment 3 months after surgery and was pain-free and back to full-time work after 5 months. A 55-year-old woman with a split depression fracture returned to her previous employment 2.5 months after surgery and was pain-free after 6 months.

**Figure 1.**
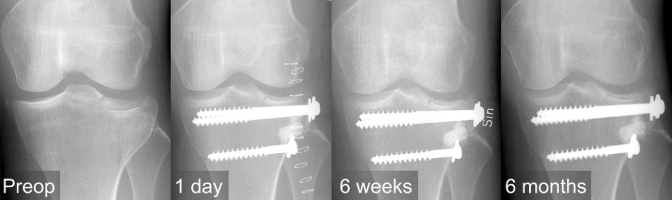
Pure depression fracture (Schatzker III) of the lateral tibial plateau in a 49-year-old woman. Follow-up radiographs showed no loss of reduction.

**Figure 2.**
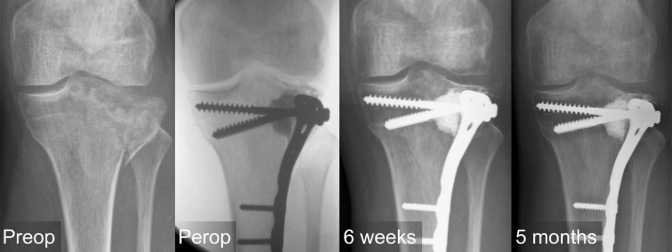
Split depression fracture (Schatzker II) of the lateral tibial plateau in a 62-year-old man. Follow-up radiographs showed no loss of reduction.

Radiographs showed anatomical reductions in all four patients, and later follow-up showed no loss of reduction (Figures 1 and 2). There were no complications or adverse effects following the use of titanium granules.

## Discussion

Porous titanium granules offer several advantages compared with autograft bone and other bone substitutes in connection surgery of compression fractures in the lateral tibial plateau: most importantly, the titanium granules are not resorbed, which means that the joint surface congruity achieved during surgery is maintained during the healing period. Further advantages over bank bone are easy access and eliminated risk of contagion. The titanium granules do not set (i.e. no risk of heat injury to the bone) and can therefore be handled without time pressure during surgery. Moreover, the fact that no bone needs to be harvested from the iliac crest means shorter surgery time and less pain for the patient.

We were impressed by the stability in the fractured area after using titanium granules. The favourable outcome in this pilot study has inspired one of us (BJ) to start a prospective randomized study, where the use of titanium granules is compared with bone grafting in the treatment of depression fractures of the lateral tibial plateau.

## References

[1] Goulet JA, Senunas LE, DeSilva GL, Greenfield ML (1997). Autogenous iliac crest bone graft. Complications and functional assessment. Clin Orthop Relat Res..

[2] Arrington ED, Smith WJ, Chambers HG, Bucknell AL, Davino NA (1996). Complications of iliac crest bone graft harvesting. Clin Orthop Relat Res..

[3] Alffram P-A, Bruce L, Bjursten LM, Urban RM, Andersson GBJ (2007). Implantation of the femoral stem into a bed of titanium granules using vibration. A pilot study of a new method for prosthetic fixation in 5 patients followed for up to 15 years. Ups J Med Sci..

[4] Turner TM, Urban RM, Hall DJ, Andersson GBJ (2007). Bone ingrowth through porous titanium granulate around a femoral stem. Histological assessment in a six-month canine hemiarthroplasty model. Ups J Med Sci..

[5] Holmberg L, Forsgren L, Kristerson L (2008). Porous titanium granules for implant stability and bone regeneration—a case followed for 12 years. Ups J Med Sci..

